# Analysis of a novel highly metastatic melanoma cell line identifies osteopontin as a new lymphangiogenic factor

**DOI:** 10.3892/ijo.2012.1548

**Published:** 2012-07-06

**Authors:** RUEDIGER LIERSCH, JAY W. SHIN, MICHAEL BAYER, CHRISTIAN SCHWÖPPE, CHRISTOPH SCHLIEMANN, WOLFGANG E. BERDEL, ROLF MESTERS, MICHAEL DETMAR

**Affiliations:** 1 Department of Medicine, Hematology and Oncology, University Hospital Muenster, D-48129 Muenster, Germany;; 2 Cutaneous Biology Research Center, Massachusetts General Hospital and Harvard Medical School, Charlestown, MA 02129, USA;; 3 Institute of Pharmaceutical Sciences, Swiss Federal Institute of Technology (ETH), 8092 Zurich, Switzerland

**Keywords:** lymphatic endothelial cells, osteopontin, lymphangiogenesis, metastasis, cancer

## Abstract

Tumor cell invasion and metastasis are hallmarks of malignancy. Despite recent advances in the understanding of lymphatic spread, the mechanisms by which tumors metastasize to sentinel/distant lymph nodes and beyond are poorly understood. To gain new insights into this complex process, we established highly metastatic melanoma cell lines by *in vivo* passaging the B16 parental cell line through the lymphatic system. In this study we characterized morphology, rate of cell proliferation, colony formation, migration, tumorigenicity, lymph flow, and capacities to induce tumor- and sentinel lymph node-lymphangiogenesis. Furthermore, microarray-based comparative analysis between parental and passaged cell lines was performed to identify specific gene expression profiles. The most differentially expressed gene was SPP (osteopontin), a secreted glycophosphoprotein which is known to be involved in cancer metastasis. Overexpression of osteopontin in B16 F1-variant was confirmed by western blot analysis and quantitative RT-PCR. Treatment of cultured lymphatic endothelial cells (LECs) with osteopontin promoted cell migration mediated by the integrin α9 pathway. Our results identify osteopontin as a novel lymphangiogenic factor.

## Introduction

Since the identification of several molecules specifically expressed in lymphatic endothelial cells a rediscovery of the lymphatic vasculature has taken place. Recent advances provided a detailed analysis of the nature and organization of the lymphatic system in physiological and pathophysiological conditions. Angiogenesis and lymphangiogenesis, the proliferation of new blood and lymphatic vessels from pre-existing ones, are characteristic features of aggressive tumors and metastasis formation. Several publications have demonstrated that the blood supply and the existence of lymphatics are of prognostic relevance (1–5). For a majority of solid tumors the metastatic involvement of the sentinel lymph node is the most important indicator for an aggressive disease, often correlating with a short overall survival (6–8). Depending on lymph node status and tumor stage, prognosis and treatment strategies may vary from more intense to less intense.

Lymphangiogenesis has been found in various malignancies. Several studies revealed that tumors can actively induce the formation of tumor lymphangiogenesis and promote metastasis (4,5,9). Dadras *et al* have shown that there is a direct relationship between lymphatic vessel density (LVD) and survival in melanoma patients (10). Furthermore, the extent of tumor lymphangiogenesis has been shown to predict the presence of melanoma metastasis in sentinel lymph nodes at the time of surgery (10). Increased LVD might be a marker of aggressive tumors that secrete high levels of lymphangiogenic cytokines. As observed by Hirakawa *et al* carcinogen-induced squamous cell carcinomas in transgenic mice overexpressing VEGF-A and VEGF-C in the skin displayed increased angiogenesis and lymphangiogenesis, as well as enhanced lymphatic and systemic metastasis (3,11). Furthermore, it has been recently described that tumors are able to induce lymphangiogenesis in sentinel lymph nodes, even prior to, and after metastatic colonization (3,11). The fact that lymph nodes respond to inflammation or neoplasia is long known. Indeed, activated lymph nodes can increase many-fold in size and weight (12–14). Although the exact mechanisms underlying lymph node lymphangiogenesis remain unclear, enhanced lymph node lymphangiogenesis could represent a possible strategy for tumors to disseminate further throughout the lymphatic system and subsequentl, to secondary sites.

So far, the main focus in targeting lymphatic endothelial cells has been the VEGFR-3 receptor. Various reports have evaluated the effect of VEGFR-3 blockade on lymphatic cancer metastasis (15). It has been shown that blocking VEGFR-3 inhibits metastasis formation, but it could not totally block metastastic spread, suggesting the existence of alternative lymphangiogenic pathways (16). We generated a highly metastatic melanoma cell line by passaging B16 cells through the lymphatic system. Morphological, pathophysiogical and molecular characteristics of the passaged B16 variants are described. In comparison to previously published B16 variants (17) these cell lines were injected intracutaneously and new variants were isolated from metastatic lymph nodes. In line with previous studies on human melanomas we observed a significant upregulation of SPP1 (osteopontin, OPN) in gene expression analyses, which is known to interact with integrin α9.

## Material and methods

### Cells

B16F1 and B16-variants were cultured in DMEM (Gibco-BRL, Grand Island, NY) supplemented with 20% fetal bovine serum (FBS). B16F1 cells (1x10^6^) were injected intradermally in the backskin of C57BL6 mice and the tumor was removed 14–17 days after implantation. Metastatic melanoma cells were isolated from lymph nodes (LN) and cultured *in vitro* (B16-RI). Then, B16-RI was again intradermally injected into mice, and primary tumors, LN metastases (B16-R2) and lung metastases (B16-R2L) were isolated and re-cultured. Funcitonal assays were performed on B16 variants *in vitro*.

### Animal models

B16 variant cells (1x10^6^) were injected intradermally in the backskin of C57BL6 mice. Tumors were measured weekly using a digital caliper and tumor volumes were calculated as published (3). After 3 weeks, mice were euthanized using carbon dioxide and tumors and lymph nodes were removed. Tissue samples were embedded in optimal cutting temperature compound (OCT; Sakura Finetek, Torrance, CA). For lymph flow evaluation we injected FITC-Dextran 500 (Sigma-Aldrich) peritumorally and lymph nodes were excised and embedded in OCT 10 min after injection.

### Matrigel assay

Wild-type mice (C57BL6, female, 6 to 8 weeks-old) were anesthesized and injected subcutaneously into the lower flank skin with 100 μl of matrigel (BD Biosciences Pharmingen, San Diego, CA) containing either human VEGF-A165 (500 ng/ml), control PBS or OPN (1,000 ng/ml; n=5 per group). After 7 days, skin samples were embedded in OCT and immunofluorescence analyses were performed on 8-μm cryostat sections, as described (18). All animal models were approved by the Massachusetts General Hospital Subcommittee on Research Animal Care.

### Quantitative real-time RT-PCR

Total cellular RNA was isolated from cultured cells using TRIzol (Invitrogen, Carlsbad, CA) and was treated with RQ1 RNase-free-DNase (Promega, Madison, WI) as previously described (19). The expression of mouse OPN mRNA was investigated by quantitative real-time RT-PCR using the ABI Prism 7000 Sequence Detection System (Applied Biosystems, Foster City, CA). Primers and probes for OPN were pre-designed (hCG38538, Applied Biosystems). Expression levels were normalized by β-actin as an internal control.

### Immunoblotting

For western blot analysis of OPN expression, supernatant of confluent cultures of B16 variants were homogenized in lysis buffer. Protein concentrations were determined using the NanoOrange Protein Quantitation Kit (Molecular Probes, Eugene, OR). Supernatants (100 μg of total protein) were then subjected to SDS-polyacrylamide gel electrophoresis using NuPAGE 10% BT gels, 1.0 mm, 12-well and NuPAGE MES SDS running buffer (Invitrogen). Proteins were transferred onto Trans-Blot Transfer Medium pure nitrocellulose membranes (BioRad, Hercules, CA) for immunoblot analysis. Blocking was performed with 5% non-fat dry milk in 0.1% Tween-20 (Sigma) in PBS, followed by immunoblotting with a polyclonal anti-mouse OPN antibody (R&D Systems, Minneapolis, MN). Specific binding was detected by the ECL Plus Western Blotting Detection System (GE Healthcare, Buckinghamshire, UK).

### Immunofluorescence and computer assisted morphometric vessel analyses

Immunohistochemical analyses were performed as previously described (18) using antibodies against mouse LYVE-1 (Upstate Biotechnology, Lake Placid, NY), CD31 (BD Biosciences Pharmingen) and corresponding secondary antibodies labeled with Alexa Fluor 488 or 594 (Molecular Probes). Cell nuclei were counterstained with Hoechst bisbenzimide (Sigma-Aldrich). Routine hematoxylin and eosin stains were also performed. Sections were examined using an Axioscope 2 mot plus (Carl Zeiss AG; Feldbach, Switzerland) and images were captured with a Zeiss AxioCam MRc. Morphometric analyses were performed using Image Pro software as described (18). Three different fields (10x) of each section were examined and the number of vessels per square micrometer, the average vessels size and the relative tissue area occupied by lymphatic vessels in the dermis were determined. The unpaired Student’s t-test was used to analyze differences in microvessel density and size.

### Proliferation, migration assays

For endothelial cell migration assays, B16 variants, lymphatic endothelial cells (LEC) or blood endothelial cells (BEC) were grown to 100% confluency and serum starved overnight. Human dermal BVEC and LEC were isolated from neonatal human foreskins, as previously described (20). Briefly, in order to remove fibrocytes, CD45-negative selection was performed using an immunomagnetic beads-conjugated anti-human CD45 antibody (Dynal, Lake Success, NY) before isolating CD34-positive BVEC. Thereafter, the remaining CD34-negative cells were incubated with an immunomagnetic bead-conjugated anti-human CD31 antibody (Dynal) to isolate LEC. Cells were washed with PBS and the medium was changed to the appropiate serum-free medium. Monolayers were incubated in 5% CO_2_ at 37°C for 24 h. For trans-well migration assays, 24-well FluoroBlock inserts of 8 μm pore size (BD Biosciences, Bedford, MA) were coated on the bottom side with 10 μg/ml fibronectin (BD Biosciences) for 1 h, followed by incubation with or without OPN (100 ng/ml) and with or without Thrombin cleavage (1 unit of Thrombin for 15 min). Protein-binding sites remaining after aspiration were blocked with 100 μg/ml bovine serum albumin (BSA; Sigma). Cells (1x10^6^/ml; 100 μl) were seeded in serum-free medium containing 0.2% delipidized BSA into the upper chambers and were incubated for 4 h at 37°C in the absence of FBS with or without blocking antibodies (α9 integrin, α1/2 integrin, Upstate Biotech, Lake Placid, NY), control-IgG antibody (10 ng/ml). Cells on the underside of inserts were stained with Calcein AM (Molecular Probes), and fluorescence intensity was measured using a Spectra Max Gemini fluorescence reader (Bucher Biotec AG, Basel, Switzerland). For proliferation assays B16 variants, LECs and BECs (2x10^3^) were seeded onto 96-well plates. Triplicate wells were treated with different concentrations of OPN (0.01 ng/ml to 2,500 ng/ml; R&D Systems) and with 20 ng/ml VEGF-A or neither. After 72 h, cells were incubated with 4-methylumbelliferyl heptanoate (MUH; Sigma-Aldrich). Fluorescence intensity, proportional to the number of viable cells was measured using a Spectra Max Gemini EM fluorescence reader (Bucher Biotec AG). Statistical analyses were performed using the unpaired Student’s t-test.

### Colony assay

Colony assays were performed according to previous reports (21). Briefly, 6-well plates were plated with semisolid medium containing MEM, 1% low-melt-agar (Roth, Karlsruhe, Germany), 10% FCS and penicillin-streptomycin. Subsequently, 5x10^4^ cells were seeded in DMEM supplemented with 10% FCS and 0.8% agarose. Dishes were monitored for 14 days and then microphotographed and colonies were counted in three high resolution fields (HRF).

### Microarray analysis

B16 and B16 variants were cultured in DMEM supplemented with 20% FBS (Invitrogen, Grand Island, NY) supplemented with antibiotic-antimycotic solution. Every B16 variant was cultured in duplicates. Total cellular RNA was isolated using the TRIzol reagent (Invitrogen, Carlsbad, CA) extracted with chloroform, precipitated with isopropanol, washed with 70% ethanol and dissolved in DNase-free/RNase-free distilled water. The concentration of RNA was measured using NanoDrop ND-1000 spectrophotometer (Witec AG, Littau, Switzerland) and RNA quality was assessed using 2100 Bioanalyzer (Agilent Technologies, Palo Alto, CA). Digoxigenin-UTP-labeled cRNA was generated and amplified from 500 ng of total-RNA using the NanoAmp RT-IVT Labeling Kit (Applied Biosystems) following the manufacturer’s protocol, and was hybridized to Applied Biosystems Mouse Genome Survey Microarrays V2.0. Chemiluminescence detection, image acquisition, and analysis were performed using the Chemiluminescence Detection Kit (Applied Biosystems) and the Applied Biosystems 1700 Chemiluminescent Microarray Analyzer following the manufacturer’s protocol. A total of two biological replicates were generated for each B16 variant RNA analysis. The data discussed in this publication have been deposited in NCBI’s Gene Expression Omnibus and are accessible through GEO Series accession number GSE33697 (http://www.ncbi.nlm.nih.gov/geo/query/acc.cgi?acc=GSE33697).

### Microarray data analysis

Raw data were normalized using the quantile normalization method available from LIMMA library - R/Bioconductor (22,23). Probes which had signal-to-noise ratio (S/N ratio) of 3 or greater, flag (error) value of 5,000 or less in duplicates for each group were further subjected to statistical analyses. Differentially expressed genes were identified using the Empirical Bayes (EB) analysis (24). The pathway analyses were performed using the PANTHER (protein analysis through evolutionary relationships) protein classification system, which classifies proteins into families/subfamilies, molecular functions, biological processes, and biological pathways (25). Pathways that were over-represented in the group of genes upregulated in new B16 variants were identified, and the significance of over-representation was calculated using binomial tests against all genes represented on the Applied Biosystems Mouse Genome Survey Microarray as the reference.

## Results

### Increased migratory properties of the new passaged cell line

**Figure 1:**
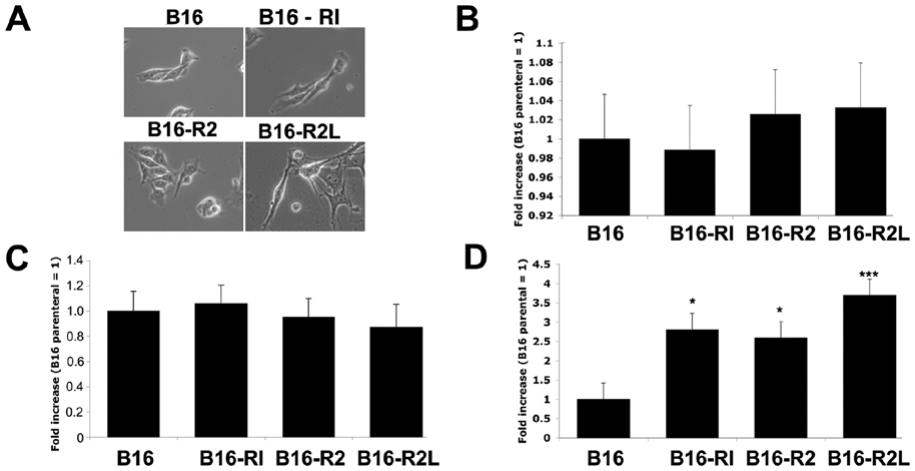
(A) Microscopy of B16-variants. Microscopic analysis of the B16-variants revealed a different morphologic cell shape and pseudopodia. Differences were maintained in late passages. New B16 variants reveal (D) an increased migration without (B) differences in proliferation and (C) colony-formation. Statistical analyses were performed using the unpaired Student’s t-test. Data represent means ± SEM. ^*^P<0.05; ^**^P<0.01; ^***^P<0.001.

**Figure 2:**
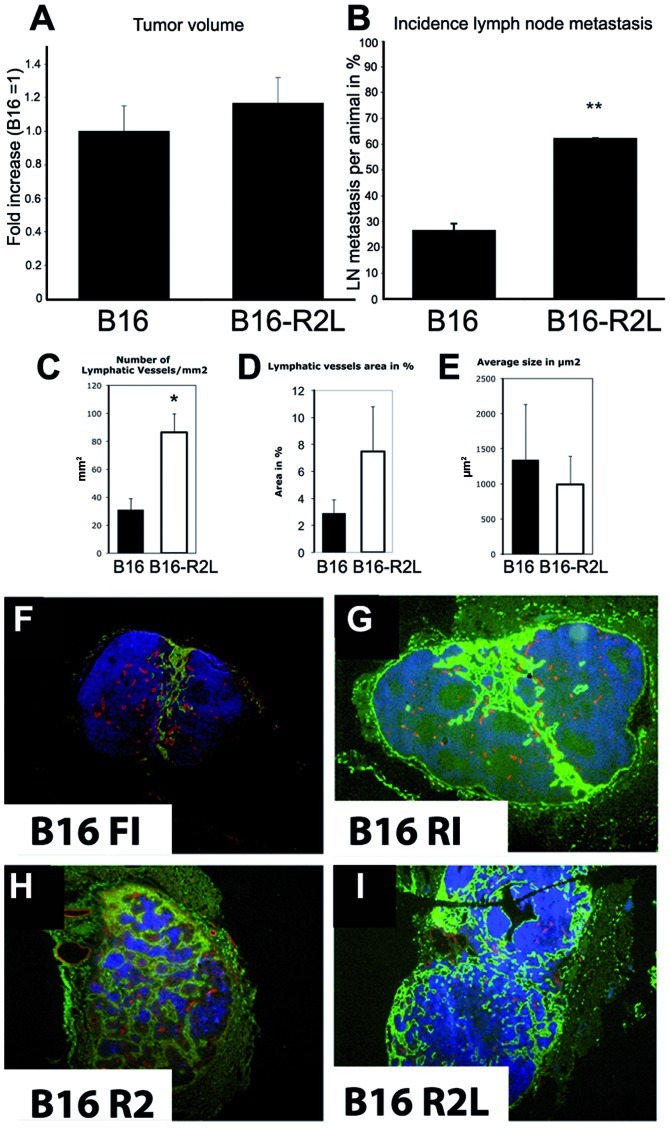
The B16-R2L variant shows (A) no difference in tumor size but (B) an increase in lymphatic metastasis to sentinel lymph nodes. B16FI and B16-R2L cells were injected intradermally (1x10^6^ in the backskin of C57Bl6) and tumor size and LN metastasis was measured/evaluated at day 14 after injection. Statistical analysis was performed using the Mann-Whitney test. Data represent means ± SEM. ^*^P<0.05; ^**^P<0.01. (C–E) Increased peritumoral lymphangiogenesis. Staining for LYVE-1 and computer-assisted evaluation (Image Pro software) reveal (C) increased peritumoral number of lymphatic vessels and (D) vessel size for the B16-R2L variant in comparison to the parental cell line (B16F1) while (E) average size was not altered. Statistical analysis was performed using the unpaired Student’s t-test. Data represent means ± SEM. ^*^P<0.05; ^**^P<0.01. (F–I) Increased sentinel lymph node lymphangiogenesis in B16 variants. Lymph node lymphangiogenesis was evaluated at day 17 after intradermal injection of 1×10^6^ cells in C57Bl6 mice (8–10 weeks). Staining revealed an increased sentinel lymph node lymphangiogenesis indicated by LYVE-1 (green)/CD31 (red) immunofluorescence stain. Nuclei, blue (Hoechst); magnification, ×5.

We first set out to investigate the *in vitro*/*in vivo* features of new B16 variant cell lines. To this aim we analyzed cell morphology, proliferation rate, colony formation, migration and tumorigenicity. Microscopic analyses revealed several key morphological changes of the new variants as compared to the parental B16 cell line (Fig. 1A). New B16 variants showed altered cell-cell junctions suggestive of increased cell motility. The B16-R2L variant especially formed incomplete cell-cell interface and revealed specific focal points on the cell surface to adhere to their neighbouring cells (Fig. 1A). The B16-F1 variant formed significantly more cell-cell interface (Fig. 1, B16), showing a typical cobblestone morphology in culture. In contrast, B16-R2L exhibited a stellate morphology under phase contrast (Fig. 1, B16-R2L), closely resembling a fibroblastic phenotype. These differences were maintained even in late passages (data not shown). While additional *in vitro* experiments revealed that the proliferation rate (Fig. 1B) and colony-forming capacity (Fig. 1C) were not altered by *in vivo* passaging, B16 variants displayed significantly increased motile propensities in migration assays (Fig. 1D). Boyden chamber migration analyses revealed a nearly 4-fold increase for B16-R2L as compared to B16F1 (Fig. 1D). To study the *in vivo* characeristics, we injected B16 parental and B16-R2L cell-line subcutaneously into the backskin of C57Bl6 mice (8–10 weeks). In agreement with *in vitro* results primary tumor growth was not altered (Fig. 2A), while lymph node metastasis, analysed by immunohistochemical analysis, was significantly increased in the new B16 variants with every passage (Fig. 2B). B16-R2L showed a nearly 2-fold higher incidence of lymph node involvement as compared to B16F1 cell line.

### Increased peritumoral lymphangiogenesis

**Figure 3:**
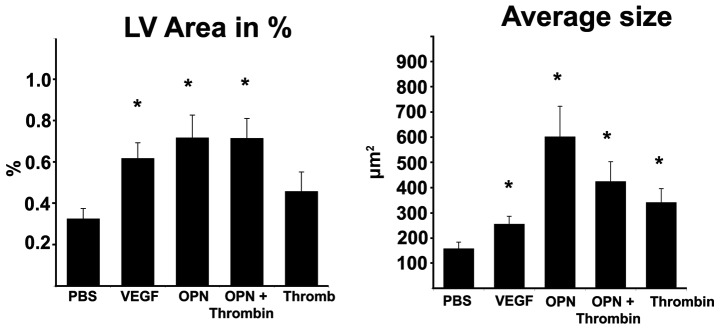
Osteopontin increases lymphangiogenesis in a Matrigel assay containing Matrigel with PBS, VEGF, OPN, OPN plus Thrombin and Thrombin. Skin was harvested after 7 days. Statistical analysis was performed using the unpaired Student’s t-test. Data represent means ± SEM; ^*^P<0.05; ^**^P<0.01.

Next, we investigated the effects of the new B16 variants on tumor-mediated formation of lymphatic and blood vessels. To analyze tumor-associated lymphatic vessels, we double-stained tumor sections with antibodies specific for mouse LYVE-1 and CD31 (Pecam). While CD31 is expressed on both endothelial cells, LYVE-1 is expressed only on lymphatic endothelial cells. Small and compressed lymphatic vessels were commonly observed in the upper skin near the tumor mass, whereas lymphatic vessels were markedly enlarged in the peritumoral area away from the skin. Intratumoral lymphatic vessels were not clearly detectable and lumina were found to be completely compressed. Computer-assisted image analysis of melanoma sections in the peritumoral regions revealed an increased number of the peritumoral-associated lymphatic vessels and percent area of enlarged vessels (Fig. 2C and D), however, the average size was not altered (Fig. 2E). These results indicate that the new B16 variants promote tumor-associated lymphangiogenesis and with only minor effects on blood vessel formation (data not shown). Finally, to investigate whether OPN alone might play a role in lymphangiogenesis *in vivo*, athymic nude mice were injected subcutaneously with Matrigel containing OPN. Lymphatic vessel area in % and average size was significantly increased surrounding the OPN-releasing implants compared with mice that received control implants (Fig. 3).

### Pronounced lymph node lymphangiogenesis and drainage by new cell line variants

**Figure 4:**
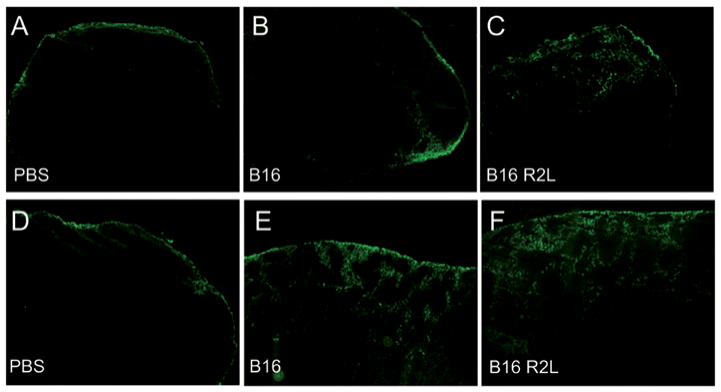
Increased cortical drainage of FITC-Dextran 500 in tumor-draining sentinel lymph nodes. PBS, B16 F1 and B16-R2L were injected intradermally in C57Bl6 mice (A–C) at day 11 and (D–F) day 15. FITC-Dextran 500 was injected peritumoral and the sentinel draining lymph node was removed after 10 min. FITC-Dextran, green; magnification, x100; representative pictures from 1 out of 4 experiments with similar outcome are shown.

Immunfluorescence analyses of lymph nodes derived from mice bearing the parental and the new cell lines revealed comparable numbers of blood vessels, while LYVE-1 positive sinusoids were strongly induced in the new B16 variants (Fig. 2 F–I). In comparison to the parental cell line, the B16 variants showed an increase of lymph sinusoids in the draining sentinel lymph nodes (Fig. 2). To determine how lymph-derived soluble factors of the lymphatic fluid are differentially drained in the sentinel lymph node of tumor bearing mice, we visualized the altered drainage of a high molecular weight protein in sentinel lymph nodes. To this end, we developed a lymph drainage analysis in sentinel lymph nodes using high molecular weight FITC-Dextran. We injected FITC-Dextran subcutaneously next to the tumor and analysed the drainage 10 min after injection in the draining lymph node. In mice bearing the new B16 variants, we could demonstrate an increased lymph flow in the cortical and subcortical zone of draining sentinel lymph nodes (Fig. 4). However, in naive mice without tumors, macromolecules were detectable in the subcapsular and medullary sinuses, but were largely excluded from the cortical lymphocyte microenvironment.

### Osteopontin as a mediator of lymphatic invasiveness

**Figure 5:**
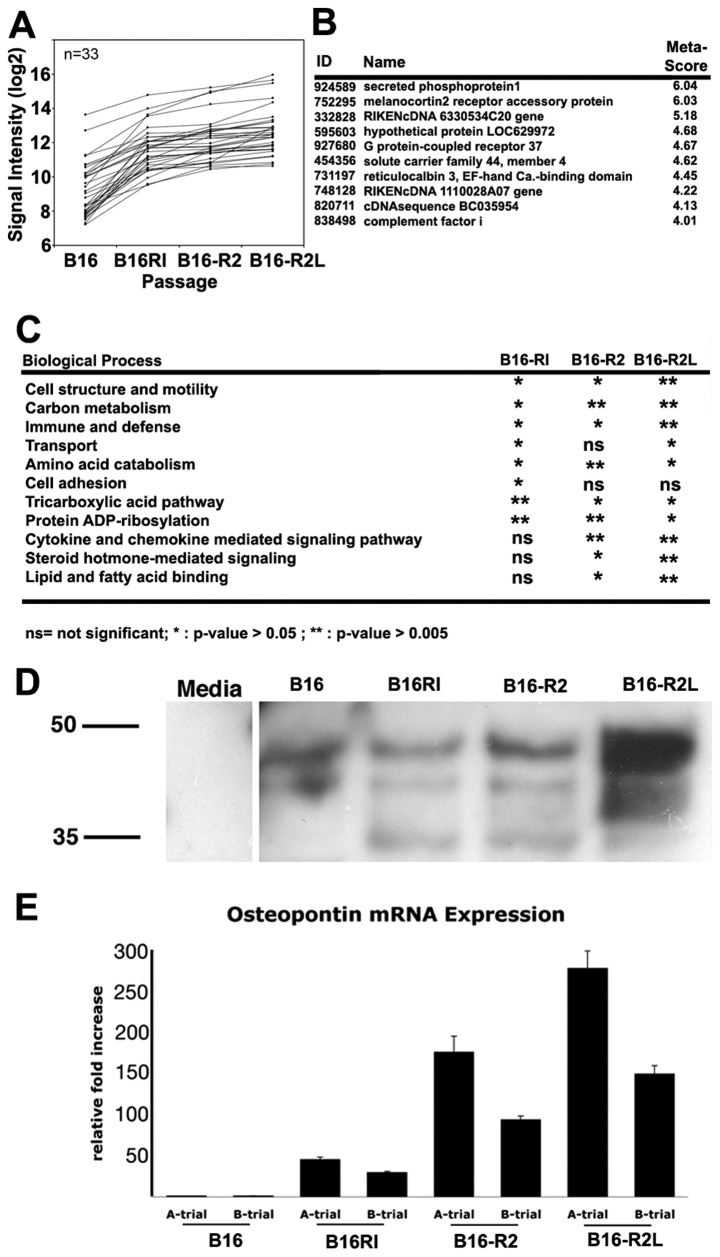
Over-expressed genes after *in vivo* passaging in an genome-wide expression array. (A) Thirty-three genes demonstrated increase in signal intensity in each passage. (B) First ten highest meta-scores are listed, (C) significantly over-represented biological processes in each *in vivo* passage. Validation of overexpressed OPN. (D) Western blot analysis of cell culture supernatant reveals secretion of OPN and (E) real-time RT-PCR indicates an increased osteopontin level in B16-variants.

To eluciate the differentital gene expression profiles of the new cell lines, we performed whole genome-wide microarray analyses. Four cell lines derived from 4 subsequent *in vivo* passages were evaluated in duplicates for microarray expression analysis. We identified 33 overexpressed genes during *in vivo* passaging and focused on those genes which where significantly upregulated with each passage (Fig. 5A and B). Analysis revealed that in the last passaged cell lines (R2 and R2L) the most significant upregulation could be seen in different biological processes (Fig. 5C). In particular, we found that the secreted phosphoprotein 1 (SPP1), also known as OPN, was significantly upregulated with *in vivo* passage (Fig. 5B). Western blots of cell line supernatants revealed the secreted OPN in B16 variants and real-time RT-PCR confirmed the microarray findings (Fig. 5D and E).

### Osteopontin directly promotes lymphatic function in vitro and in vivo

**Figure 6:**
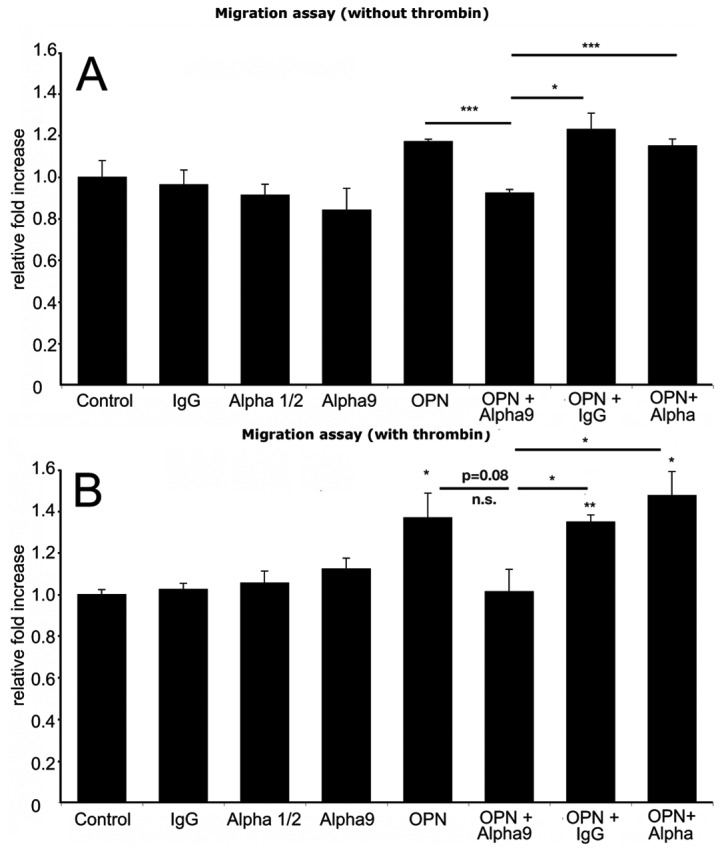
OPN increases LEC migration. OPN, especially after (B) thrombin cleavage, induces LEC migration. This process could be blocked by the anti-α9 integrin antibody. Statistical analysis was performed using the unpaired Student’s t-test. Data represent means ± SEM. ^*^P<0.05; ^**^P<0.01.

We investigated whether OPN might directly stimulate wound-repair associated lymphangiogenesis via activation of α9 integrin, which is known to be expressed by lymphatic vessels. While the treatment of cultured human LEC with OPN induced no significant increase of LEC migration in a haptoxis assay in comparison to the control cells the antibody blockade of α9 integrin significantly inhibited LEC migration to a lower level observed in OPN stimulated and unstimulated LECs (Fig. 6A). This shows that OPN without thrombin cleavage already has an effect on α9 integrins, which could possibly be explained by other proteases. Blockade of either the α1 or the α2 integrin subunit had no significant impact on OPN-induced LEC migration (Fig. 6A). Considering the thrombin cleavage effect, we repeated the experiments with thrombin cleavage revealing that the observed effect could be enhanced by OPN processing, especially in contrats to the untreated cells, while in comparison to α-antibody, cell treatment lacked significance value (P=0.08; Fig. 6B).

## Discussion

In many tumors, the prognosis and the treatment decisions change depending on lymph node status and tumor stage. Once tumor cells gain access to the lymphatic system it has been proposed that lymphogenous and hematogenous metastases can occur almost simultaneously (26). Recently, a new mode of tumor lymphangiogenesis has been described by Hirakawa *et al* observing an increased lymphangiogenesis in the sentinel lymph node, even prior to, and after metastatic colonization (3,11). In addition, it has been recently documented that lymphatic sinus growth is associated with greatly increased lymph flow within lymph nodes in melanoma-bearing mice and developing B-cell lymphomas (27,28). The results presented here support these observations by a comparison of different *in vivo* passaged melanoma cells lines with differential lymphatic invasiveness. In line with the results of Hirakawa *et al* we found no metastasis in distant organs without lymph node metastasis (11).

Furthermore, our results indicate that sentinel lymph node lymphangiogenesis might increase the drainage of metastasing cancer cells into the deeper cortex, facilitating metastasis either through the lymphatic system or rapidly through the circulatory system. Recent publications by Rudell *et al*(28) revealed that lymphatic sinuses express VEGFR-2 and VEGFR-3 and that subcutaneously injected dyes travel more efficiently through draining lymph nodes, indicating that hyperthropic lymphatic sinuses increase lymph flow. However, the drainage pattern of injected macromolecules has not been described. It is tempting to speculate that tumor cells could get better access into the lymph node due to these structural changes in draining lymph nodes. These intriguing findings reveal that different metastatic cell lines induce lymphangiogenesis within the draining lymph nodes even before the tumor has metastasized, possibly facilitating future metastatic spread via the lymphatic and blood vessel system. Whether this effect is exclusively mediated by OPN is not clear.

OPN is a phosphorylated secreted chemotactic protein of the SIBLING family that is known to attract immune cells to inflammatory sites. The protein is expressed at high levels in a variety of tumors and transformed cells (29–32) and expression can either be localized in the tumor cells themselves (30,32) or in tumor-associated macrophages (29). Several studies indicated OPN expression in various human malignancies, acting as a marker of malignancy in prostate cancer, osteosarcoma, glioblastoma, squamos cell carcinoma, breast cancer and melanoma (reviewed in ref. 33). In melanoma, it has been demonstrated that OPN inhibition reduces melanoma cell growth (34) and decreases B16 melanoma invasiveness (35). In our analysis we confirmed that OPN is abundantly expressed in metastatic melanomas, and these cancer cells are even characterized by differential behaviour *in vitro* and *in vivo*. OPN overexpression results in an increase in the malignant phenotype (36), a finding which has generally been observed in metastatic melanomas (37). It has been reported that transfection with antisense oligonucleotides reduced the malignant potential of malignant melanoma (38), overexpression of OPN in OPN-negative breast cancer cells increased metastasis through a mechanism that enhances angio-genesis (39). In contrast, we did not observe an increase of the tumor volume between the different variants and angiogenesis in peri- and intratumoral areas in our *in vivo* experiments. However, we demonstrate for the first time that OPN is a strong stimulator of lymphatic endothelial cell migration and lymphangiogenesis. The difference in the average size of the lymphatic vessels in the two different *in vivo* experiments could be explained by the different tissue pressure and time-point of analysis.

Various factors are known to be involved in the regulation of lymphatic endothelial cells and recently it has been shown that integrins such as α1β1, α2β1, α5, αv and also α9 are expressed by lymphatic endothelial cells (40). The integrin α9 recognizes at least three distinct ligands: vascular cell adhesion molecule 1 (VCAM-1), tenascin-C and OPN. Smith *et al* showed that a recombinant OPN fragment could support α9β1-mediated adhesion, an activity not found in the full-length molecule (41). In addition, they found that α9β1 could mediate melanoma cell migration (41). The proteolytic fragments of OPN play a potential role not only on tumor migration itself, but also on lymphatic endothelial cell migration and therefore tumor-derived OPN facitiltates the future metastasis not only by direct mechanism on the melanoma cell itself, but also by activation of lymphatic endothelial cells. We have found that OPN can induce lymphatic cell migration and that it is secreted in greater quantity by lymphatic invasive melanomas. Furthermore, our results indicate that OPN-induced lymphatic cell movement is a directed, α9β1-dependent response. Cell proliferation studies have shown that the major effects of OPN involve lymphatic migration but not proliferation.

Since its first identification in bone, the role of OPN has been an area of intense investigation. OPN plays a crucial role in determining the metastatic potential of different cancer types in various ways. The expression of OPN confers a survival advantage in tumor cells. In summary, our results indicate a potential role of OPN in migration of lymphatic endothelial cells, as an insoluble adhesive ligand. OPN, which is expressed at high levels in many tumor types, is functioning as a cell adhesion molecule in the extracellular matrix. Owing to its potential role in stimulating cancer cell motility, tumor growth and metastasis, OPN might be considered as a promising candidate target for the treatment of cancer.
